# Hypolocomotion, asymmetrically directed behaviors (licking, lifting, flinching, and shaking) and dynamic weight bearing (gait) changes are not measures of neuropathic pain in mice

**DOI:** 10.1186/1744-8069-6-34

**Published:** 2010-06-08

**Authors:** Jeffrey S Mogil, Allyson C Graham, Jennifer Ritchie, Sara F Hughes, Jean-Sebastien Austin, Ara Schorscher-Petcu, Dale J Langford, Gary J Bennett

**Affiliations:** 1 Dept. of Psychology and Alan Edwards Centre for Research on Pain, McGill University, Montreal, QC H3A 1B1 Canada; 2 Dept. of Anesthesia, Faculty of Dentistry and Alan Edwards Centre for Research on Pain, McGill University, Montreal, QC H3A 1B1 Canada

## Abstract

**Background:**

Spontaneous (non-evoked) pain is a major clinical symptom of neuropathic syndromes, one that is understudied in basic pain research for practical reasons and because of a lack of consensus over precisely which behaviors reflect spontaneous pain in laboratory animals. It is commonly asserted that rodents experiencing pain in a hind limb exhibit hypolocomotion and decreased rearing, engage in both reflexive and organized limb directed behaviors, and avoid supporting their body weight on the affected side. Furthermore, it is assumed that the extent of these positive or negative behaviors can be used as a dependent measure of spontaneous chronic pain severity in such animals. In the present study, we tested these assumptions via blinded, systematic observation of digital video of mice with nerve injuries (chronic constriction or spared nerve injury), and automated assessment of locomotor behavior using photocell detection and dynamic weight bearing (i.e., gait) using the CatWalk^® ^system.

**Results:**

We found no deficits in locomotor activity or rearing associated with neuropathic injury. The frequency of asymmetric (ipsilaterally directed) behaviors were too rare to be seriously considered as representing spontaneous pain, and in any case did not statistically exceed what was blindly observed on the contralateral hind paw and in control (sham operated and unoperated) mice. Changes in dynamic weight bearing, on the other hand, were robust and ipsilateral after spared nerve injury (but not chronic constriction injury). However, we observed timing, pharmacological, and genetic dissociation of mechanical allodynia and gait alterations.

**Conclusions:**

We conclude that spontaneous neuropathic pain in mice cannot be assessed using any of these measures, and thus caution is warranted in making such assertions.

## Background

The perception that basic pain research over the last two decades has not always resulted in clinical advances has encouraged reflection as to how animal models of pain may be improved [1]. Proposals include the use of operant instead of reflexive dependent measures [2], the collection of a broader range of measurements besides pain behaviors *per se *[3], and the measurement of behaviors spontaneously emitted by the rodent subject [4]. The latter is important because in neuropathic pain patients, spontaneous (continuous or paroxysmal) pain is thought to be the most prevalent pain related symptom, the most bothersome, and the most highly correlated with overall pain ratings [5,6]. Whether or not drugs differentially affect spontaneous and stimulus-evoked pain in the clinical setting is a subject deserving of much more attention than it has received (e.g., [7]).

Various behaviors following injuries in rodents are purported to be real time measures of spontaneous pain. For example, there are a few published reports of ultrasonic vocalizations during inflammatory pain [8,9], but two systematic investigations including nerve injuries concluded that ultrasonic vocalization is not specifically related to pain [10,11]. Other candidates are: 1) hypolocomotion (both horizontal and vertical; that is, walking and rearing); 2) asymmetric directed behaviors including biting, flinching, licking, lifting, scratching and/or shaking of the ipsilateral hind limb; and, 3) guarding of the affected limb, leading to weight bearing and gait changes [4]. In a number of papers, the total amount of such behaviors observed in neuropathic animals is used as a measure of spontaneous pain intensity (e.g., [12-18]). Conceptually, it is not clear that all of these behaviors relate to spontaneous pain. For example, guarding the hind paw while walking might be better thought of as pain-avoidance behavior. However, in contrast to stimulus-evoked withdrawal reflexes, these behaviors may be more realistic models of a patient's everyday pain experience.

Assertions that a given behavior is a real-time measure of spontaneous pain should be examined more critically. Observation periods in these studies are usually quite brief, often lasting only 5-10 min. Rarely are the sessions videotaped and archived, allowing for more detailed analysis and public inspection. Observations are often made very soon after the nerve injury, rendering it unclear whether observed behaviors reflect neuropathic or postoperative pain. Until recently, most relevant data have been collected in the laboratory rat, but the mouse continues to gain popularity as a subject for basic pain research [1].

With respect to the measurement of weight bearing, many popular techniques require either restraint [19] or the animal being forced to maintain an unusual standing position over force plates [20]. The study of arthritic patients has suggested that dynamic weight bearing measures are more clinically relevant than static measures because walking aggravates the pain. Furthermore, most static measures of weight bearing involve only the hind limbs, and injury to the hind limbs can shift weight distribution to the fore limbs [21]. A recently developed technique to measure dynamic weight bearing is the CatWalk^® ^system (Noldus Inc.), and one study performed on rats observed significant correlations between mechanical allodynia and CatWalk-measured gait changes after nerve injury [22].

In the current study, we evaluate the frequency, timing and asymmetry of some of these measures after nerve injuries (chronic constriction injury, spared nerve injury) in the laboratory mouse. In addition, we demonstrate pharmacological and genetic dissociation of mechanical hypersensitivity (as measured by von Frey fibers) and proposed spontaneous measures. We conclude that these measures are all, for one reason or another, not appropriate for the measurement of spontaneous neuropathic pain in the laboratory mouse.

## Results

### Absence of sex differences

All experiments featured male and female subjects in approximately equal numbers. No main effects of sex were noted in any experiment. A statistical interaction involving sex was observed in only one experiment, the 22 strain SNI study (see below), in which von Frey-measured allodynia and CatWalk intensity changes both featured a significant genotype × sex interaction (*p *< 0.005 in both cases). This interaction is not relevant to the current topic, however, and thus data from both sexes were combined for all analyses reported below.

### Mechanical allodynia in mice given CCI

**Figure 1:**
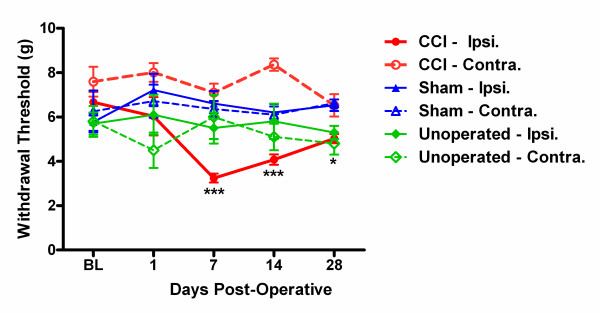
**Ipsilateral but not contralateral mechanical allodynia in CD-1 mice given CCI (red)**. No significant changes from baseline von Frey thresholds were seen in either hind paw after sham surgery (blue) or simple repeated testing in unoperated mice (green). Symbols represent mean ± S.E.M. withdrawal threshold (average of two determinations per hind paw) from a nylon filament applying slowly increasing force (Ugo Basile Dynamic Plantar Aesthesiometer) to the plantar surface of the hind paws, measured before surgery (baseline; BL), and 1, 7, 14 and 28 days postoperatively. *p < 0.05 compared to baseline and contralateral side; ***p < 0.001 compared to baseline and contralateral side.

To confirm the adequacy of our CCI surgeries, a separate cohort of mice (*n *= 8) were given surgeries and tested for mechanical allodynia (automated von Frey test) preoperatively, and at the same time points used for video analysis, 1, 7, 14 and 28 days post-surgery. Results, shown in Fig. 1, reveal significant and robust allodynia of the ipsilateral hindpaw (*F*_3,21 _= 9.2, *p *< 0.001 by repeated measures ANOVA), reaching a maximum at days 7 and 14 and showing signs of amelioration by day 28. In CD-1 mice, CCI produces no contralateral allodynia (*F*_3,21 _= 1.5, *p *= 0.25). Mice receiving sham surgeries and unoperated mice displayed no significant changes from baseline in either hind paw (all *p*'s > 0.05). Highly similar data have been obtained in our laboratory previously using the same surgeon (J. S.A.) and strain, but with conventional (i.e., manual) von Frey fibers using the up down method (data not shown).

### Locomotor activity in mice given CCI

**Figure 2:**
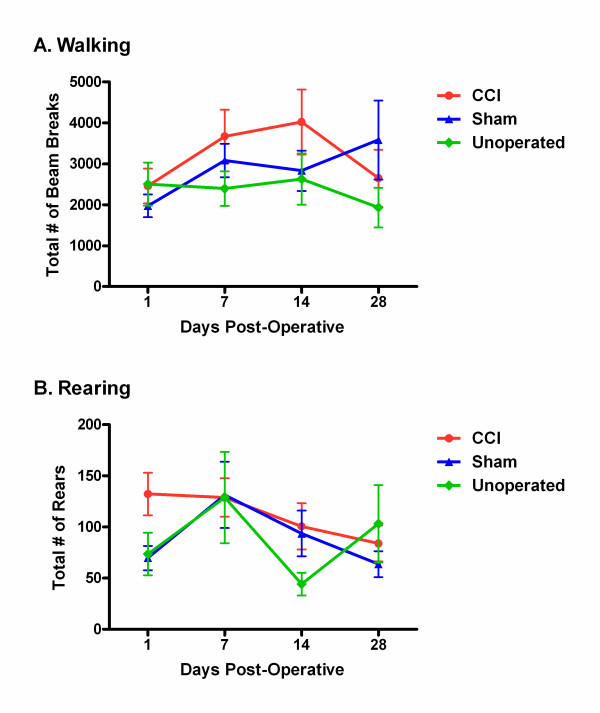
**No evidence for reduced locomotor behavior in CCI mice**. Symbols represent mean ± S.E.M. (A) walking (total beam breaks minus repetitive breaks of the same beam) and (B) rearing on the hind paws (total breaks of beams located ≈ 10 cm above the floor) in 60 min of mice given CCI surgeries (red), sham surgeries (blue), or unoperated (green), and tested 1, 7, 14 and 28 days post-operatively.

Walking and rearing behavior were assessed using the automated system. As can be seen in Fig. 2, no significant differences between CCI, sham and unoperated groups were detected overall, or on any post-operative day. For walking--defined as the total number of photocell beam breaks minus repetitive breaks of the same beam--repeated measures ANOVA revealed no main effect of group (*F*_2,15 _= 1.4, *p *= 0.28), repeated measure (*F*_3,45 _= 2.0, *p *= 0.14), nor group × repeated measure interaction (*F*_6,45 _= 1.6, *p *= 0.16). For rearing--defined as total number of breaks of photocell beams located ≈ 10 cm above the floor--no main effect of group (*F*_2,23 _= 1.1, *p *= 0.34), repeated measure (*F*_3,69 _= 1.8, *p *= 0.16), nor group × repeated measure interaction (*F*_6,69 _= 0.9, *p *= 0.50) was observed.

### Frequency of asymmetric behaviors in mice given CCI

**Figure 3:**
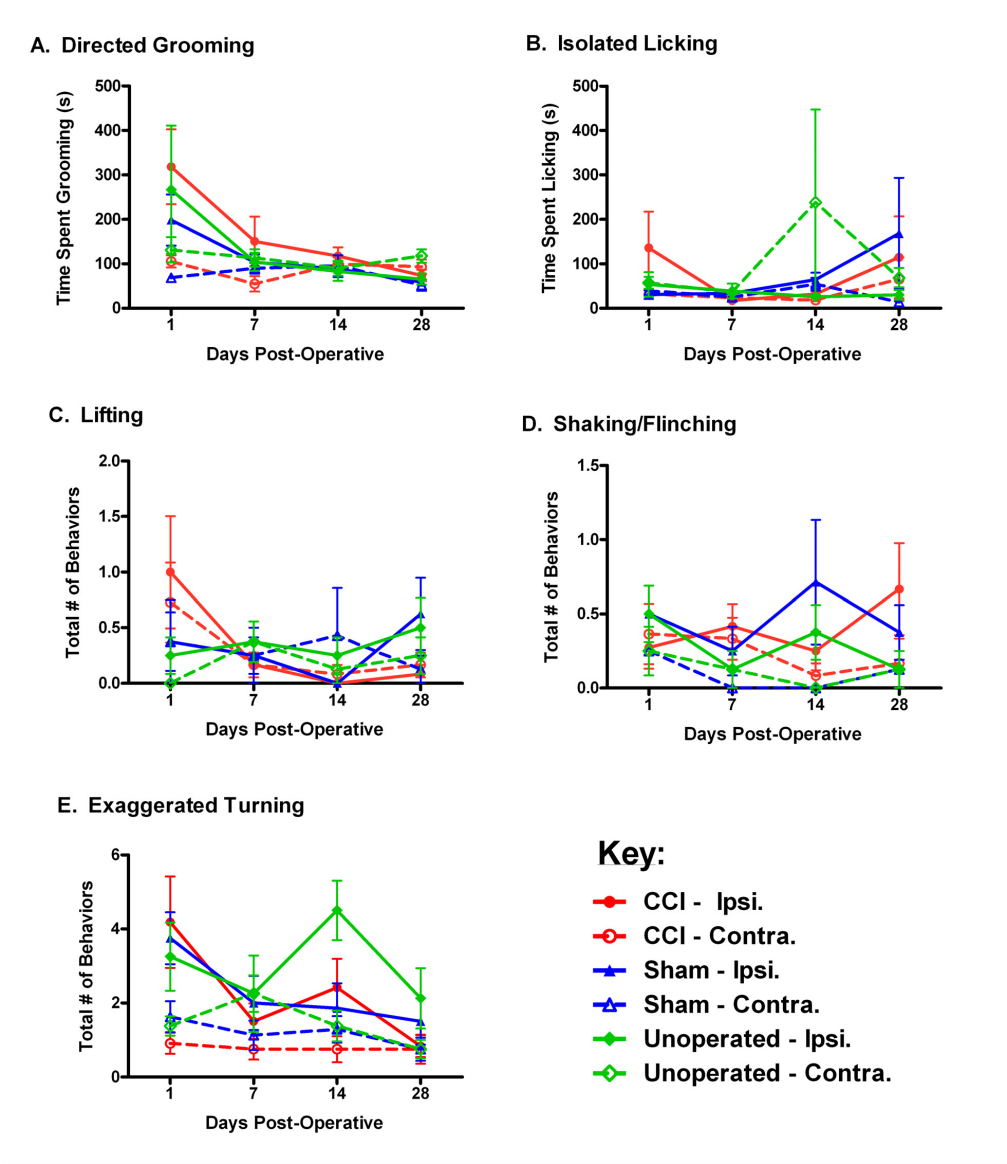
**No evidence for asymmetrically directed behaviors in CCI mice**. Symbols represent mean ± S.E.M. (A) directed grooming behavior (duration in s), (B) isolated licking behavior (duration in s), (C) hind paw lifting behavior (counts), (D) shaking/flinching behavior (counts), and (E) exaggerated turning behavior (counts) in 60 min of mice given CCI surgeries (red), sham surgeries (blue), or unoperated (green), and tested 1, 7, 14 and 28 days post-operatively. Closed symbols and solid lines (see key in bottom right) refer to the ipsilateral (Ipsi) side; open symbols and dashed lines refer to the contralateral (Contra) side. Definitions of all behaviors can be found in the main text; example video clips are available as Additional files 1, 2, 3, 4 and 5. Full videos are available upon request.

Analysis of 60-min videos made at each post-operative time point in all experimental groups is shown in Fig. 3. Each measure was analyzed via a two-way (surgical condition, side) repeated measures ANOVA. For directed grooming (Fig. 3A), a significant main effect of side was observed (*F*_1,46 _= 5.8, *p *= 0.02), as was a significant effect of repeated measure (*F*_3,138 _= 8.6, *p *< 0.001) and a significant repeated measure × side interaction (*F*_3,138 _= 7.0, *p *< 0.001). This reflects the higher levels of grooming on post operative day 1 on the ipsilateral side in all groups, and can be interpreted simply as reflecting attention being paid to the shaved ipsilateral flank. The fact that unoperated mice groom as much as sham-operated mice suggests that grooming does not represent post-operative pain from the surgery. For isolated licking (Fig. 3B) and lifting (Fig. 3C), no main effects nor interactions were observed. For shaking/flinching (Fig. 3D), a significant main effect of side was observed (*F*_1,46 _= 6.9, *p *< 0.05), reflecting slightly higher levels of this behavior in the ipsilateral hind paws in all surgical conditions. Finally, for exaggerated turning (Fig. 3E), a significant main effect of side (F_1,46 _= 28.9, *p *< 0.001) and main effect of repeated measure (*F*_3,138 _= 4.5, *p *< 0.005) were observed. This reflects the generally (but unevenly) higher turning to the ipsilateral side compared to the contralateral side in all conditions.

Importantly, in no case did we observe a significant condition × side interaction in the absence of a significant main effect of repeated measure, or a significant condition × side × repeated measure interaction (nor any trend towards either), as would be demanded by any useful dependent measure of neuropathic pain in this experiment. That is, in no case did we see evidence of these behaviors being more frequent in CCI mice. Also, all behaviors were in fact extraordinarily rare. Grooming and licking behavior were typically observed for a total of 50-100 seconds, representing <3.0% of the total scoring time. Hindpaw lifting and shaking/flinching were observed less than 0.5 times per hour on average, and exaggerated (ipsilateral) turns only 2-4 times per hour.

### von Frey and CatWalk changes after SNI in 22 mouse strains

**Figure 4:**
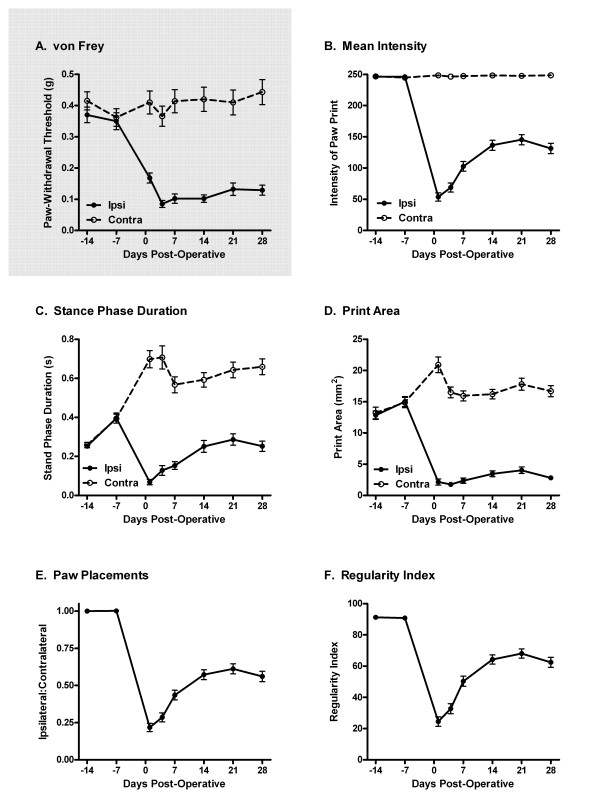
**Dissociation of time course of mechanical allodynia and dynamic weight bearing (gait) changes following SNI injury**. Data are combined from 22 inbred mouse strains (see text). Symbols represent mean ± S.E.M. 50% withdrawal threshold from von Frey filaments (g; in A), and mean ± S.E.M. CatWalk gait parameters including: (B) mean paw print intensity (arbitrary scale from 0-250), (C) stance phase duration (s), (D) paw print area (mm^2^), (E) paw placement ratio (ipsilateral: contralateral), and (F) regularity index (arbitrary scale from 0-100). For definitions see main text. In all graphs, data from the ipsilateral hindpaw is shown using closed symbols and solid lines; where relevant, data from the contralateral hindpaw is shown using open symbols and dashed lines. No significant changes in forepaw parameters were seen (data not shown). Note that mechanical allodynia peaks at post-operative day 4 and remains statistically unchanged thereafter, whereas CatWalk gait parameters display maximal changes at post operative day 1 and then partially recover. Individual significance levels are not shown for clarity; owing to the very large sample size (*n *= 122) all statements above are supported at *p *< 0.001 by repeated measures ANOVA.

Mechanical allodynia as measured by conventional von Frey testing was observed in each of the 22 mouse strains. In addition, robust changes in all CatWalk parameters were seen in each strain. However, these changes could be dissociated both in terms of their time courses and their strain-dependence. Fig. 4 shows the time course of von Frey withdrawal thresholds and the five CatWalk parameters in the complete data set, irrespective of strain (*n *= 123-127/measure). As can be seen, von Frey allodynia was present on post operative day 1, peaked on day 4, and remained statistically equivalent thereafter all the way to day 28 (Fig. 4A; all *p*'s < 0.001). Sham-operated mice displayed no significant changes in either von Frey withdrawal thresholds or CatWalk parameters, even on post-operative day 1 (data not shown). For every CatWalk parameter maximum changes were seen at day 1 (in contrast with the day 4 peak for allodynia), and in every case statistically significant improvement was seen after that point, reaching a plateau at approximately day 21 (Fig. 4B-F; all *p*'s < 0.001). Improvement in all parameters except print area represented approximately half of the initial change on post operative day 1.

**Figure 5:**
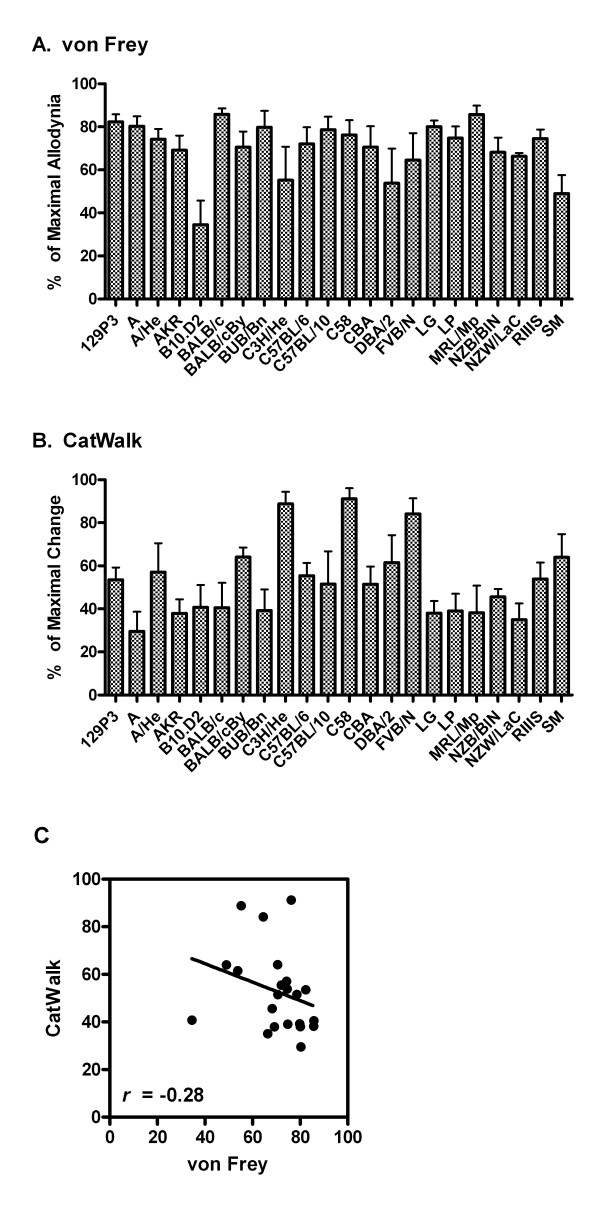
**Genetic dissociation between mechanical allodynia and dynamic weight bearing (gait) changes following SNI injury in 22 inbred mouse strains**. Bars represent mean ± S.E.M. (A) percentage of maximal allodynia and (B) percentage of maximal weight bearing (mean paw print intensity; see Fig. 4B) change, in each strain separately, over 28 days post-operatively, calculated as areas over the time-effect curve using the trapezoidal rule. Robust strain differences (*p *< 0.001) were obtained for both measures; the individual pattern of strain responses is the subject of a manuscript in preparation. The non-significant correlation between these two measures is shown in graph C; symbols represent individual strains. Note that the trend towards a negative correlation would argue *against *genetic variability in allodynia and gait changes being produced by similar gene variants.

There were significant main effects of strain in *overall *von Frey and CatWalk changes over the 28-day time course, calculated as areas over the time effect curve using the trapezoidal rule (*p*'s < 0.001). Strain means of all CatWalk parameters were highly inter-correlated (*r *= 0.67-0.97, all *p*'s < 0.01). Correlations of all other CatWalk parameters with mean intensity were all *r *≥ 0.76 (*p *< 0.001), and so we used this parameter--featuring a completely stable baseline and no potentially confounding post surgical alterations whatsoever in any other paw--to represent CatWalk changes by strain. Fig. 5 shows strain-dependent total von Frey allodynia of the ipsilateral hind paw (Fig. 5A), CatWalk total mean intensity decreases in the ipsilateral hind paw (Fig. 5B), and their correlation (Fig. 5C). As can be seen, the correlation between von Frey and CatWalk changes was a non-significant *r *= -0.28 (*p *= 0.20), and it should be noted that the trend is in the *opposite *direction to that which would indicate they measure the same underlying phenomenon. This conclusion is not confounded by body weight or willingness to cross the runway (and thus average walking speed), since although these parameters were highly strain dependent both at baseline and in their changes after neuropathic injury (data not shown), no correlations of these parameters with any CatWalk measures even approached significance. It should be noted that Clarke and Still [23], in their seminal analysis of gait in the mouse, demonstrated that all major gait variables were consistent over a wide range of velocities.

No CatWalk measures were altered significantly from baseline at any post-surgery time point in CD-1 mice (*n *= 11) given CCI surgeries (data not shown). This is not due to genotype differences, since CD 1 mice displayed highly significant changes in gait as measured by CatWalk after SNI (see below). Why SNI would produce guarding and CCI would not is not immediately obvious, but it should be noted that Piesla and colleagues [24] demonstrated differences in the gait changes produced by various nerve injuries (and inflammatory compounds) in the rat. In their hands, however, CCI in rats clearly produces changes in gait, suggestive of a rather striking species difference.

### CatWalk changes do not respond to analgesics

**Figure 6:**
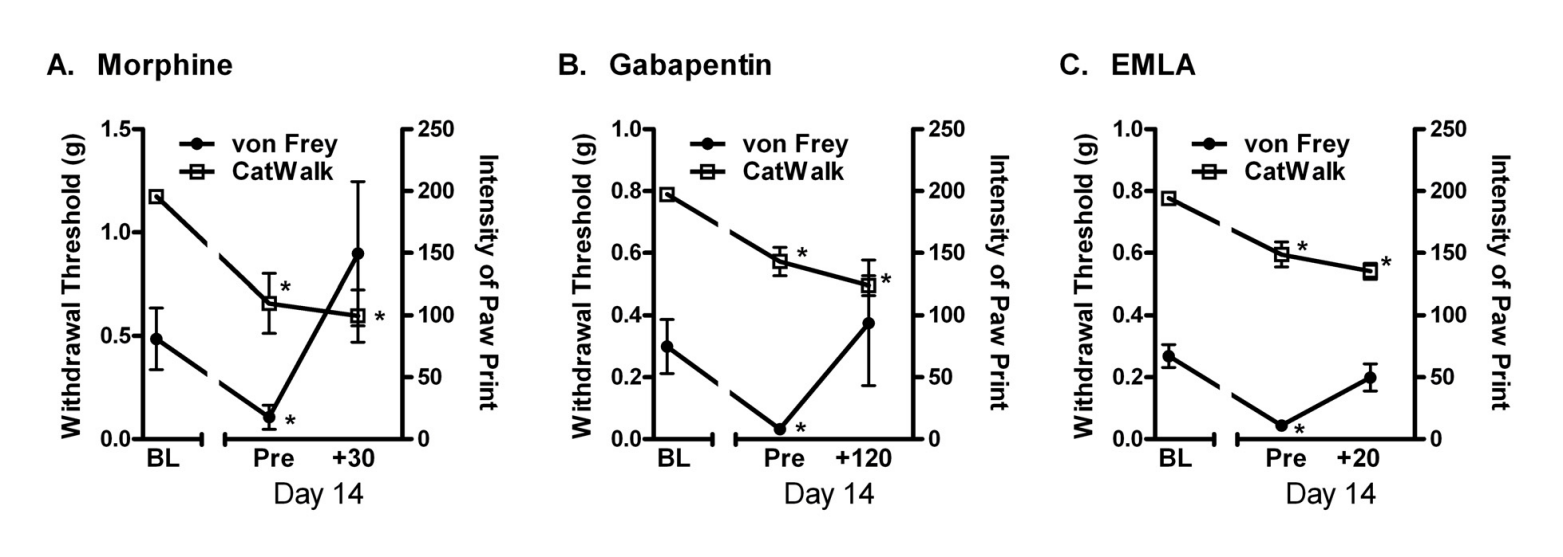
**Pharmacological dissociation between mechanical allodynia and dynamic weight bearing (gait) changes following SNI injury in CD-1 mice**. Symbols in A-C represent mean ± S.E.M. von Frey withdrawal thresholds (g; solid symbols) and CatWalk ipsilateral hind paw print intensity (0-250; open symbols) prior to SNI (baseline; BL) and on post-operative day 14, both prior to (Pre) and subsequent to (time post-injection in min is listed) administration of 10 mg/kg morphine (A), 75 mg/kg gabapentin (B), or topical EMLA (C). *p < 0.05 compared to same-test BL.

On day 14 post-surgery, robust ipsilateral mechanical allodynia on the von Frey test and significant CatWalk hind paw print intensity decreases were observed in each drug group (Fig. 6A-C; all *p*'s < 0.005), replicating in CD-1 mice what was previously observed in the 22 inbred strains. Repeated-measures ANOVAs were performed separately on von Frey and CatWalk data for each drug. Morphine, gabapentin and EMLA all produced efficacious anti allodynia, restoring von Frey thresholds to baseline (pre operative) levels, and in the case of morphine, beyond (all *p*'s < 0.05 compared to day 14 pre-injection baseline by repeated measures ANOVA). However, at the same doses and time points, none of the three drugs significantly altered CatWalk mean intensity values (*p*'s = 0.60, 0.10, and 0.10, respectively, compared to day 14 pre injection baseline, with trends towards *decreased *weight bearing on the ipsilateral hindpaw) or any other CatWalk parameter (data not shown). That is, morphine, gabapentin and EMLA reversed mechanical allodynia produced by the neuropathic injury, but did not affect the gait abnormalities also associated with that injury. Pilot studies (data not shown) confirmed that morphine, gabapentin and EMLA produced no changes in any CatWalk parameters by themselves (i.e., in the absence of injury). Non significant trends towards increases in von Frey withdrawal thresholds of unoperated mice were produced by morphine (baseline: 0.50 ± 0.08 g;

30 min post injection: 0.64 ± 0.21 g) and gabapentin (baseline: 0.43 ± 0.30 g; 120 min post-injection: 1.10 ± 0.27 g), but not EMLA (baseline: 0.70 ± 0.28 g; 20 min post injection: 0.65 ± 0.28 g).

## Discussion

An optimal measure of spontaneous pain would have a number of characteristics:

1) The measure should be *specific *to pain, and not an indicator of stress, illness, emotional/motivational changes, or cognitive (i.e., attention, learning, memory) or motor dysfunction. Although chronic neuropathic pain in humans is often associated with these phenomena, and they represent important facets of the pain experience in humans, analgesic development efforts generally require that the biological system being affected by the compound be well defined. Specificity is also critical so that the phenotypic effects of genetic mutations can be interpreted. 2) The measure should be displayed with high enough *frequency *such that partial analgesic effects of drugs or genetic alterations can be discerned. This requirement is both theoretical and practical; the face validity of very rare behaviors is suspect, but even if valid, experiments using the measure might nonetheless be seriously hampered by "floor effects." 3) In assays featuring unilateral injuries the measure should be *asymmetric*, appearing significantly more frequently on the ipsilateral than the contralateral side. Although "mirror pain" is well known [25], contralateral changes are always more modest than ipsilateral changes in animal models. 4) The measure should be *objective*, with high inter- and intra-rater reliability. 5) *Empirical (predictive) validity *ought to be established by demonstrating the efficacy, against the measure, of known analgesics of multiple chemical classes.

Additional practical considerations in favor of adopting a measure of spontaneous pain include *ease *(and/or automation) of data collection and *singularity *(i.e., one measure, not a composite of many), for clarity of analysis and interpretation. Finally, for many purposes, an optimal measure would have the property of *immediacy*, reflecting the existence of spontaneous pain in real time, rather than measuring the consequences of a chronic pain state integrated over time (e.g., anxiety). In this respect, *positive *behaviors are better than negative (i.e., reduction of normal) behaviors.

We would note that one spontaneous behavior related to neuropathic pain fulfilling all these criteria actually exists: autotomy (self-mutilation) behavior in the neuroma model [26]. Its usage remains limited, however, based on aesthetic considerations (i.e, the presence of blood), continued debate over what percept is causing the behavior, and the rarity of the behavior in partial nerve injury models thought to be most relevant to common neuropathic pain disorders.

### Asymmetrically directed spontaneous behaviors

We find no statistical evidence of any hypolocomotion or spontaneously emitted behaviors directed at the presumed site of pain (i.e., the ipsilateral hind paw) in nerve injured mice that exceed levels observed in sham-operated or unoperated mice. Because of the possibility of mirror pain [25], comparison to sham-operated animals may be more appropriate than comparison to the contralateral hindpaw. To remain consistent with standard procedures in the field, we tested all mice in their light cycle. However, a pilot experiment in which neuropathic mice were tested for 6 hours overnight (i.e., during their active phase) under red illumination only also revealed no trend whatsoever towards ipsilateral predominance of spontaneously emitted behaviors (data not shown). We note that although sample sizes in these experiments were not exceptionally large (*n *= 8-12/measure), they equal or exceed the median sample size of *n *= 8 used in modern pain research [27]. We note that it remains possible that small subsets of mice might display such behaviors, just as only a minority of humans receiving nerve damage go on to develop chronic pain syndromes (see [28]), but even if this is true as a practical matter these dependent measures would not be valuable in pain research experiments featuring typical sample sizes.

**Figure 7:**
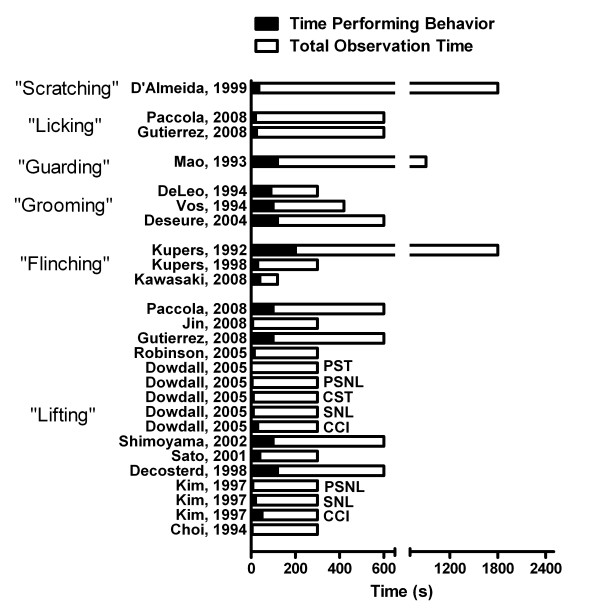
**Frequency of proposed behaviors indicating chronic spontaneous pain in the neuropathic pain literature**. Bars indicate total duration (s) of the behavior (filled) and total observation time (open). All studies used surgical nerve injuries, which are not noted except in studies featuring multiple surgeries. Note that the "scratching" behavior quantified by D'Almeida et al. [16], first described in Kupers et al. [18], is defined as "a rapid vibration of the hind paw," and is probably analogous to what is defined herein as shaking/flinching. Abbreviations: CCI, chronic constriction injury; CST, complete sciatic transection; PSNL, partial sciatic nerve ligation; PST, partial sciatic transection; SNL, spinal nerve ligation. References (from top; only first author noted): D'Almeida, 1999 [16], Paccola, 2008 [61], Guttierez, 2008 [62], Mao, 1993 [63], DeLeo, 1994 [64], Vos, 1994 [65], Deseure, 2004 [66], Kupers, 1992 [67], Kupers, 1998 [18], Kawasaki, 2008 [12], Jin, 2008 [68], Robinson, 2005 [69], Dowdall, 2005 [53], Shimoyama, 2002 [70], Sato, 2001 [71], Decosterd, 1998 [72], Kim, 1997 [52], and Choi, 1994 [73].

In an attempt to compare the current data to the wider literature, a literature search was performed (PubMed search terms "spontaneous pain" AND [mouse OR rat] AND [chronic OR neuropathic]", plus papers already known to the last author), revealing approximately 50 relevant papers that were studied in further detail. What is striking from these studies is the brevity of the observation period (only two studies exceeding 15 min), the multiple instances where behaviors were asserted but not quantified, the comparisons to baselines, contralateral hind paws, or unoperated controls instead of sham operated animals, and the great variability encountered in the frequency/duration of the behaviors themselves. In an attempt to compare apples-to-apples, Fig. 7 displays data from 17 studies using neuropathic pain models that explicitly quantified the total duration of the behavior. As can be seen, these behaviors are rare, comprising a median percentage of 10% of total observation time. It is possible that the even less-frequent observation of these behaviors in the present study is due to the more conservative definitions that we used. We note that the frequency of some of these same asymmetrically directed behaviors in models of inflammatory and especially cancer pain are sometimes reported to be much higher (e.g., [29-32]), raising the possibility that neuropathic pain models are fundamentally different in this respect.

### Gait changes

In marked contrast to our findings with respect to asymmetric behaviors, we observed robust alterations in gait (i.e., weight bearing during locomotion) produced by SNI (but not CCI). Such changes have been interpreted either as evidence of spontaneous pain and/or guarding; that is, attempts by the animal to avoid the mechanical allodynia that would result from hind paw to-floor contact [33]. In fact, weight bearing changes have been proposed as a confound of mechanical allodynia measurement via von Frey fibres [34]. Direct evidence that gait changes represent pain or guarding (to avoid pain) is somewhat sparse. Using incapacitance testing to measure static (i.e., standing) weight bearing in inflammatory models, a variety of analgesic manipulations appear efficacious [35]. With respect to dynamic weight bearing measured by the CatWalk and its predecessors, effects on arthritic changes have been shown with buprenorphine [36], muscimol [37], morphine [38,39] and cyclooxygenase inhibitors [38,39]. However, we are unaware of any prior evaluation of the effects of known analgesics on dynamic weight bearing changes caused by nerve injury. In fact, to our knowledge only one study has directly tested CatWalk changes in a common animal model of neuropathic pain. In CCI rats, Vrinten and Hamers [22] observed that the time course of CatWalk changes paralleled that of von Frey measured mechanical allodynia, and that correlations (in individual rats) between von Frey and various CatWalk measures ranged from *r *= 0.61 to 0.67. The contrast with present data might be explained by species or pain model differences, but it should also be noted that their sample size was *n *= 12 whereas ours is *n *= 122.

In the present strain survey experiment, we demonstrated that there is no genetic correlation whatsoever between von Frey allodynia and any CatWalk measure, suggesting that these are independent phenomena [40]. The independence of allodynia and gait is reinforced by the time course of changes, which although similar in general terms are actually quite different on closer inspection, with von Frey allodynia still clearly developing when gait alterations are at their maximum, and von Frey allodynia remaining constant over the 28 day testing period while gait recovers considerably over the same time span. Finally, the existence of allodynia but not concomitant gait alterations after CCI in the mouse argues for the independence of these phenomena.

It is entirely possible that all these data simply point to the fact that spontaneous and evoked pain are mechanistically distinct (see ref. [50]). However, the fact that three separate analgesic manipulations at doses that were entirely successful at reversing allodynia were wholly unable to affect gait strongly suggests that gait changes may not be related to pain at all. Of interest is a very recent study in the rat by Piesla and colleagues demonstrating that gabapentin and duloxetine reverse mechanical hyperalgesia after nerve injuries but have no effect on gait parameters measured using the DigiGait™system [24]. In their hands, some gait abnormalities produced by carrageenan were normalized by morphine and indomethacin. With respect to knee joint antigen-induced arthritis in rats, one recent study concludes that some gait parameters represent pain but others are indices of mechanical joint deformation [41], and another observed only transient gait changes [42].

Weight bearing and gait may be altered independently of whether the animal is experiencing spontaneous pain after nerve injury. In the SNI and other surgical procedures motor axons that innervate the muscles of the paw and lower leg are damaged [43]. Na and colleagues [44] concluded that the postural abnormalities seen in the SNI model were partially due to this motor deficit. The ventroflexion of the toes, loss of toe spreading, and eversion of the hind paw seen after CCI in rats (but not in mice; unpublished observations) are likewise due to motor axon damage. Other possibilities include tethering of the nerve to the adjacent muscle due to scar tissue formation [45]. Damaged axons are mechanosensitive [46] and it is possible that the animal is trying to avoid the sensations that are produced by stretching the nerve.

## Conclusions

The current observations lead to one of a few possible conclusions. First, it is possible the CCI and SNI neuropathic states studied here simply don't produce spontaneous pain, at least in the mouse. Second, it is possible that the surgeries do produce spontaneous pain in mice, but that none of the spontaneously emitted behaviors measured here are accurate measures of that pain. Finally, it is possible that spontaneous chronic pain either does not exist (i.e., all pain is evoked) or does not lead to *any *measurable behaviors in mice. Mice, as a prey species, have obvious motivations to hide signs of vulnerability from potential predators and even conspecifics. Certain noxious stimuli (e.g., formalin, bee venom, capsaicin, mustard oil) do produce obvious and robust spontaneously emitted behaviors in mice. However, longer-lasting inflammatory stimuli (e.g., carrageenan, zymosan, complete Freund's adjuvant) generally do not, and as the present study makes clear, neither does nerve damage.

## Methods

### Animals

Subjects in all experiments but one were naïve, adult (7-12 week old) outbred CD 1^® ^(ICR:Crl) mice of both sexes [47], bred in our laboratory from mice obtained from Charles River (Boucherville, QC). Mice were weaned at 18-21 days and housed with their same sex littermates. In one experiment, adult mice of both sexes from 22 inbred mouse strains (129P3, A, A/He, AKR, B10.D2-H2/oSn, BALB/c, BALB/cBy, BUB/Bn, C3H/He, C57BL/6, C57BL/10, C58, CBA, DBA/2, FVB/N, LG, LP, MRL/Mp, NZB/BIn, NZW/LaC, RIIIS, and SM; all "J" substrains), were obtained from The Jackson Laboratory (Bar Harbor, ME), and habituated to the vivarium for at least one week before testing. Mice were housed in standard shoebox cages, 2-4 per cage, maintained in a temperature-controlled (20 ± 1°C) environment (14:10 h light cycle; with lights on at 07:00 h), and fed (Harlan Teklad 8604) and watered ad libitum. All procedures were approved by a McGill University animal care and use committee and were consistent with Canadian Council on Animal Care guidelines.

### Experimental nerve injuries

After baseline testing for mechanical sensitivity (in some experiments; see below), all mice received unilateral surgical nerve injuries under isoflurane/oxygen anesthesia using either the chronic constriction injury (CCI) model [48] or the spared nerve injury (SNI) model [49], as adapted for the laboratory mouse [50,51]. Sham CCI surgeries were conducted identically except that no ligations were placed around the sciatic nerve. Sham SNI surgeries were conducted identically except that no nerves were cut. Unoperated mice were housed, handled and tested identically to other groups (including anesthesia and shaving the flanks), but received no surgeries. Because both Kim et al. [52] and Dowdell et al. [53] suggested that CCI in the rat produces the strongest evidence of spontaneous pain (defined as hind paw lifting) of multiple neuropathic injury models studied [also see [54]], most of the current experiments were conducted using the CCI model. The gait analysis experiment was performed using the SNI model, since data from this experiment were being collected originally for other purposes, and since no evidence of gait changes were observed in mice after CCI surgeries (data not shown).

### von Frey testing

Mice were placed individually in transparent Plexiglas cubicles (5 cm wide × 8.5 cm long × 6 cm high; each cubicle was separated from the other by an opaque divider to abolish "neighbour effects" [55]) placed upon a perforated metal floor (with 5 mm diameter holes placed 7 mm apart), and habituated for 2 h before behavioral testing began. In one study, nylon monofilaments (Stoelting Touch Test Sensory Evaluator Kit #2 to #9; calibrated weekly using a microbalance; ≈ 0.015, 0.04, 0.07, 0.15, 0.44, 0.55, 1.0 and 1.3 g) were firmly applied to the plantar surface of the hindpaw (alternating the side of the body being tested) until they bowed for 5 s. Only withdrawal responses performed obviously in response to the applied stimulus were scored. The up-down method of Dixon [56] was used to estimate 50% withdrawal thresholds. In other studies, an automated von Frey test (Ugo Basile Dynamic Plantar Aesthesiometer) was employed. In the CCI model, von Frey fibers were applied to the mid-plantar hind paw. In the SNI model, we spared the sural nerve and thus applied von Frey fibers to the lateral aspect of the plantar hind paw to measure mechanical allodynia. Mice were only tested when alert or resting [57]. At each time point, two separate threshold determinations were made on each hind paw, and then averaged.

### Locomotor activity measurement

We measured horizontal (walking) and vertical (rearing) locomotor activity of mice given CCI surgeries, sham surgeries, or no surgery (unoperated) (*n *= 6/condition).

Mice were singly housed within standard shoebox cages with an aerated Plexiglas cover (to reduce hanging behavior, which confuses the software), habituated for 30 min, and then monitored for 60 min via a fully automated, photocell-based system (Opto Varimex Micro Animal Activity System™; Columbus Instruments; Columbus, OH). Sessions occurred at 1, 7, 14 and 28 days post-surgery, all at 19:30 h (± 1 h). The decision to test animals near the beginning of their dark (active) phase was made to decrease the probability of sleeping, but mice were tested with normal room lighting (30 lux) to better approximate current procedures in the field.

### Video analysis in cubicles

So as not to bias the observation, an undergraduate researcher (A.G.) not familiar with the neuropathic pain literature was instructed simply to observe, unblinded, multiple videos of neuropathic mice (CCI model; *n *= 8), and identify behaviors seeming potentially significant to her (data not shown). She identified: hindpaw licking (both that occurring as part of the normal, stereotyped grooming sequence [58], and also that isolated from it), hindpaw lifting, hindpaw shaking/flinching, and what she termed "exaggerated turning" to the side of the injury. Definitions are as follows, and video clips illustrating these behaviors are available to interested researchers:

*Directed Grooming*: Grooming of the haunch, knee, ankle or paw, excluding isolated licking (see below). Measured as total duration of behavior (s).

*Isolated Licking*: Licking of the toes or footpad of the hind paw, neither immediately preceded or immediately followed by licking of any other part of the body. Measured as total duration of behavior (s).

*Lifting*: Holding the hind paw aloft in a manner not obviously associated with locomotion, rearing, licking, or body repositioning. Measured as number of discrete events.

*Shaking/Flinching*: Shaking of the hind limb (usually high-frequency).

Measured as number of discrete events.

*Exaggerated Turning*: An abrupt and pronounced turn of the head, in an apparent attempt to examine the hind limb. Measured as number of discrete events.

Once these behaviors were decided upon, videos including unoperated mice and mice given sham surgery were interspersed with those of new neuropathic mice (*n *= 8-12/condition), and scored blindly by the same observer. To preserve blinding, unoperated mice had one of their flanks shaved to resemble those of operated mice. CD 1 mice were placed in cubicles as described above, atop a 1/4-inch-thick glass floor. After a 30-min habituation period, their behavior was recorded from below with high resolution video cameras for 60 min. The digital video files were archived, and scored later using Noldus Observer™ 5.0 or LabSpy™ software. One-hour-long videos of each mouse were made at 1, 7, 14 and 28 days post-surgery, all at 19:30 h; unoperated mice were videotaped simultaneously.

### CatWalk testing

This experiment was added to a separate study being performed simultaneously, in which 22 inbred mouse strains were tested for mechanical allodynia as measured with von Frey monofilaments for the purpose of identifying neuropathic pain variability genes via haplotype mapping (manuscript in preparation). CD-1 mice were not tested in this experiment as they are outbred, and thus not appropriately included in a strain survey aimed at identifying genetic determinants. Mice (*n *= 4-9/strain) were baseline tested twice, at one week intervals, prior to surgery (SNI model), and on post-operative days 1, 4, 7, 14, 21 and 28. On each testing day, mice were tested on the CatWalk as described below, and then habituated and tested for mechanical allodynia.

The CatWalk^® ^system (Noldus Inc.) of automated gait analysis has been described in detail previously [59]. Subjects traverse a walkway (50.5 cm long; 3.3 cm wide) atop a glass floor in a darkened room. Light enters the distal long edge of the glass floor from a fluorescent bulb located at the side, and is internally reflected, scattering only at points where a paw touches the glass, producing bright illumination of the contact area. Internal reflection is incomplete, allowing a faint superimposed image of the animal to be seen as well. A video camera monitors the corridor, and the digitized signal is stored for later analysis of animals crossing the walkway.

A number of "single-paw" and interlimb coordination parameters can be obtained; we found the following to be the most reliable in the mouse:

1. *Mean Intensity*. The average intensity of the pixels at the maximum paw-floor contact, a proxy measure of the relative force being exerted on the floor by that paw (i.e., favoring).

2. *Stance Phase Duration*. Time of contact of the paw with the glass floor, another measure of favoring.

3. *Print Area*. Total surface area of the glass floor contacted by the paw during the complete stance phase, which would be decreased if the animal were attempting to avoid placing a certain part of the plantar hindpaw on the floor.

4. *Paw Placements*. Number of placements of each paw on the walkway. Can be used to calculate the ratio of ipsilateral to contralateral hind paw placements. This parameter will detect attempts to avoid placing a hind paw on the floor entirely.

5. *Regularity Index*. The degree of interlimb coordination during gait, expressed as the percentage of total paw placements conforming to one of three normal step sequence patterns, including "alternate", "cruciate" and "rotary" [60].

These parameters were calculated for each paw in each mouse on each testing day. Mice were allowed (and, if necessary, encouraged by prodding) to cross the CatWalk three times on each testing day, with results averaged. Possible confounds of the CatWalk are body weight and motivation to cross the walkway (leading to changes in velocity). Thus, on each testing day, body weight was measured, and the experimenter assigned a score to each mouse indicating its "willingness" to cross (3: mouse moves down runway smoothly with no need of prodding; 2: mouse needs slight prodding but then moves down runway smoothly; 1: mouse needs constant prodding to get it to move; 0: mouse freezes, climbs up side of runway wall, or attempts to turn around or back up).

Mechanical allodynia or CatWalk guarding behavior (measured by mean intensity) over the entire 28-day testing period was calculated for each mouse as the area-over-the curve using the trapezoidal rule. Percentages of maximum possible allodynia and guarding were calculated by comparing observed areas-over-the-curve to the maximum possible for each individual mouse considering its own baseline.

### Drug challenge experiments

Naïve CD-1 mice were tested for baseline von Frey sensitivity or on the CatWalk, and then given surgeries (SNI model) as described. In this experiment, separate groups of mice (*n *= 6-12/drug/test) were used for von Frey and CatWalk measurements. On post-operative day 14, they were tested again on the von Frey or CatWalk test immediately before and after systemic injection of morphine (10 mg/kg, s.c.) or gabapentin (75 mg/kg, s.c.), or after application of EMLA™ cream (eutectic mixture of 2.5% lidocaine, 2.5% prilocaine), spread in a 2-mm layer over the entire plantar hind paw in mice anesthetized for 45 min with isoflurane/oxygen. Owing to the different time courses of peak effect of these analgesic manipulations (based on pilot experiments; data not shown), mice were tested 30 min after morphine, 120 min after gabapentin, and 20 min after the EMLA was wiped off the hind paw of the awakening mouse (at which point mice were completely recovered from the anesthesia). Previously collected data in our laboratory (not shown) confirmed an absence of sedation/ataxia on the rotarod test produced by 10 mg/kg morphine or 75 mg/kg gabapentin.

Separate groups of unoperated mice (*n *= 8/drug) were simply administered the three analgesics and tested for von Frey withdrawal thresholds and on the CatWalk to see if they produced any effects on mechanical sensitivity or dynamic weight bearing *per se*.

### Statistical analyses

Data were analyzed by ANOVA or Student's *t*-test, as appropriate. A criterion significance level of α = 0.05 was adopted in all cases. A small number (*n *= 6) of statistical outliers (>3 S.D.) not developing mechanical allodynia were removed from the analysis in the SNI strain survey data set. In four cases, locomotor activity data was not collected by the software on one testing day.

## Competing interests

The authors declare that they have no competing interests.

## Authors' contributions

JSM conceived of the study, directed the experiments and wrote the manuscript. ACG, JR, SFH, JSA, ASP and SJL carried out the experiments. GJB helped design the study and edited the manuscript. All authors have read and approved the final manuscript.

## Acknowledgements

This work was supported by an unrestricted gift from the Louise and Alan Edwards Foundation (JSM). We thank Dr. Chris Flores for helpful discussions.

## Supplementary Material

Additional file 1 **Directed Grooming**. Click here for file

Additional file 2 **Isolated Licking**. Click here for file

Additional file 3 **Paw Lift**. Click here for file

Additional file 4 **Paw Shake**. Click here for file

Additional file 5 **Exaggerated Turn**. Click here for file
